# Notch1 Pathway Activity Determines the Regulatory Role of Cancer-Associated Fibroblasts in Melanoma Growth and Invasion

**DOI:** 10.1371/journal.pone.0142815

**Published:** 2015-11-12

**Authors:** Hongwei Shao, Ranran Kong, Massimiliano L. Ferrari, Freddy Radtke, Anthony J. Capobianco, Zhao-Jun Liu

**Affiliations:** 1 Department of Surgery, University of Miami School of Medicine, Miami, Florida, United States of America; 2 Ecole Polytechnique Fédérale de Lausanne, Swiss Institute for Experimental Cancer Research, Lausanne, Switzerland; 3 Department of Thoracic Surgery, the 2nd Affiliated Hospital, Xi’an Jiaotong University School of Medicine, Xi’an, China; Vanderbilt University, UNITED STATES

## Abstract

Cancer-associated fibroblasts (CAF) play a crucial role in regulating cancer progression, yet the molecular determinant that governs the tumor regulatory role of CAF remains unknown. Using a mouse melanoma model in which exogenous melanoma cells were grafted on the skin of two lines of mice where the genetic activation or inactivation of Notch1 signaling specifically occurs in natural host stromal fibroblasts, we demonstrated that Notch1 pathway activity could determine the tumor-promoting or tumor-suppressing phenotype in CAF. CAF carrying elevated Notch1 activity significantly inhibited melanoma growth and invasion, while those with a null Notch1 promoted melanoma invasion. These findings identify the Notch1 pathway as a molecular determinant that controls the regulatory role of CAF in melanoma skin growth and invasion, unveiling Notch1 signaling as a potential therapeutic target for melanoma and potentially other solid tumors.

## Introduction

CAF are stromal fibroblasts residing within and in the vicinity of the tumor mass. They are primarily derived from activated local quiescent fibroblasts and recruited circulating bone marrow mesenchymal stem cells (MSC) [1,2]. CAF are involved in regulating tumor progression by eliciting soluble factors, extracellular matrix (ECM) [3] and exosomes [4]. Their contribution to primary and secondary malignancies [5,6] as well as taking part in drug resistance and tumor recurrence [7,8] make CAF potential targets for therapeutic interventions on the tumor microenvironment (TME). Despite extensive evidence supporting the crucial tumor-regulating role of CAF, how the role is determined remains a mystery. We and others previously observed that Notch1 pathway activity is inversely correlated with that of fibroblasts. Notch pathway activity is low in proliferating fibroblasts, while high in quiescent fibroblasts [9]. Loss of *Notch1* in mouse embryonic fibroblasts (MEF) resulted in faster cell growth and motility rate, whereas Notch1 activation retarded cell growth and motility of human fibroblasts [9]. Consistently, Notch activation induced cell-cycle arrest and apoptosis in MEF [10]. In tumor xenograft mouse models, co-implanted experimental human dermal fibroblasts carrying high Notch1 activity inhibited melanoma growth and angiogenesis [11], demonstrating that Notch1 activation confers a tumor-suppressive phenotype on experimental CAF. These results suggested a crucial role for Notch signaling in governing function of fibroblasts. However, the fibroblasts investigated in these earlier studies are not real or natural CAF. Here we utilized novel mouse models to explore the role of Notch1 signaling in determining the regulatory role of natural host CAF in melanoma growth and invasion.

## Materials and Methods

### Mice

*Notch1* ^*Loxp/LoxP*^ mice were described [12]. *ROSA* ^*LSL-N1IC+/+*^ (#008159) and *Fsp1*.*Cre* ^*+/-*^ (#012641) mice were purchased from The Jackson Lab (Bar Harbor, ME). All these mice have a C57BL6 background. The Gain-Of-Function Notch1 (GOF^Notch1^: *Fsp1*.*Cre* ^*+/-*^;*ROSA* ^*LSL-N1IC+/+*^) and Loss-Of-Function Notch1 (LOF^Notch1^: *Fsp1*.*Cre* ^*+/-*^;*Notch1* ^LoxP/LoxP+/+^) lines were generated by crossing *ROSA* ^*LSL-N1IC+/+*^ and *Notch1* ^*Loxp/LoxP+/+*^ with *Fsp1*.*Cre* ^*+/-*^ mice, and subsequently crossing *Fsp1*.*Cre* ^*+/-*^;*ROSA* ^*LSL-N1IC+/-*^ with *ROSA* ^*LSL-N1IC+/+*^ mice and *Fsp1*.*Cre* ^*+/-*^;*Notch1* ^LoxP/LoxP+/-^ with *Notch1* ^*Loxp/LoxP+/+*^ mice, respectively. GOF^ctrl^ (*FSP1*.*Cre* ^*-/-*^ *;ROSA* ^LSL-N1IC+/+^) and LOF^ctrl^ (*FSP1*.*Cre* ^*-/-*^ *; Notch1* ^LoxP/LoxP+/+^) mice were used as control. Mice were maintained at the DVR animal facility under standard conditions. Mice were anesthetized for all surgical procedures by ketamine/xylazine mixture (100/10 mg/kg, IP), and imaging procedures by inhaling 3% isoflurane gas, and sacrificed in CO2 chamber. Institutional animal care and use committee at the University of Miami approved all animal procedures.

### Mouse skin model of melanoma

Murine melanoma cells, B16-F10 (ATCC^®^, CRL-67345^TM^), stably transduced with Luciferase 2 (Luc2)/lentivirus, were cultured with complete DMEM. For tumor graft experiments, 5 x 10^5^ Luc2^+^/B16-F10 cells suspended in 0.1 ml saline were inoculated (*s*.*c*.*)* on dorsal skin of 6-week old GOF^Notch1^ vs. GOF^ctrl^ and 8-week old LOF^Notch1^ vs. LOF^ctrl^ mice. B-16-F10 are derived from C57BL6 mouse and can be xenografted on the created GOF, LOF and control mice which have a C57BL6 background. The mice were sacrificed at week 3 after grafting. Resected tumors were weighted. Melanoma growth was assessed based on tumor weight and positivity of Ki67 cell proliferation marker measured by immunofluorescence in tumor cells, while melanoma local invasion was evaluated by histological assessment of tissue sections of resected melanoma.

### Histology, Immunofluorescence (IF) & Western blot

H&E and IF were performed as described [11]. Antibodies recognize activated Notch1, Hes1, Ki67, Luc (ab8925, ab71559, ab15580, ab81823, Abcam, Cambridge, MA), Hey-1, and FSP1 (GTX42614, GTX89197, GeneTex, Irvine, CA). Nuclei were stained with DAPI (Sigma-Aldrich, St. Louis, MO). Quantifications are mean ± standard deviation (SD) of counts from 5 low power field (LPF) per tumor sample for H&E staining, and 5 high power field (HPF) per section and 5 section/tumor for IF staining. For Western blot, skin tissue samples were homogenized in a RIPA buffer (50 mM Tris-Cl, 150 mM sodium chloride 1.0% NP-40, 0.5% sodium deoxycholate, 0.1% SDS, pH 8.0, 1 mM EDTA and protease inhibitor cocktails (Roche)). The tissue suspension was rotated at 4°C for 30 min. Supernatants were collected after centrifugation at 13,000 rpm for 15 min. Concentration of protein was determined using Pierce^TM^ BCA Protein Assay Kit (#23225). Western blot was conducted as described [11]. Expression of mutant N1^IC^ (muN1^IC^, 59Kd) and deletion of Notch1 in mouse skin were detected by two different anti-Notch1 antibodies, respectively (ab8925 and ab52627, Abcam).

### Bioluminescence imaging of IVIS

D-luciferin was injected intra-peritoneally 15 minutes prior to imaging (150mg/kg). Whole-body of anesthetized mice were scanned using IVIS 200B (PerkinElmer, Waltham, MA) with a 1 minute capture, medium binning. Bioluminescence signal was quantified and reported as total light emission within the region of interest (photon/s).

### Lentivirus and cell transduction

Luc2^+^/lentiviral vector was constructed by inserting *Luc2* cDNA into *pLenti6* (Invitrogene) vector. Production of lentivirus and transduction of cells were performed as described [13].

### Statistical analysis

The data were statistically analyzed using two-tailed Student’s *t*-test and expressed as the mean ± SD. The values are considered statistically significant when *p*<0.05.

## Results

### Notch1 activation in CAF suppresses melanoma growth

Expression of fibroblast-specific protein-1 (FSP1, also called S100A4) is generally restricted to fibroblasts [14,15]. *FSP1*.*Cre* mice were successfully used to create null alleles in fibroblasts for TGFβ type II receptors [16], EP4 receptors [17], and PTEN [18]. We created the 1^st^ pair of GOF^Notch1^ vs. GOF^ctrl^ mice. GOF^Notch1^ mice were viable until employed in melanoma skin graft experiments within two months. Their body appearance and skin tissue histology, including dermis where skin fibroblasts primarily reside, appeared normal (S1A Fig).

To examine the role of CAF with high Notch1 activity in regulating melanoma growth, 5 x 10^5^ Luc2+/B16-F10 cells were inoculated onto skin of GOF^Notch1^ vs. GOF^ctrl^ mice. In this model, the cellular components of entire tumor stroma, including CAF, are composed of natural host cells. Tumor metastasis was monitored by whole-body IVIS scanning at week 3 post tumor inoculations. Skin tumors were resected, weighted and subjected to immunohistochemical analysis after IVIS scanning.

**Fig 1:**
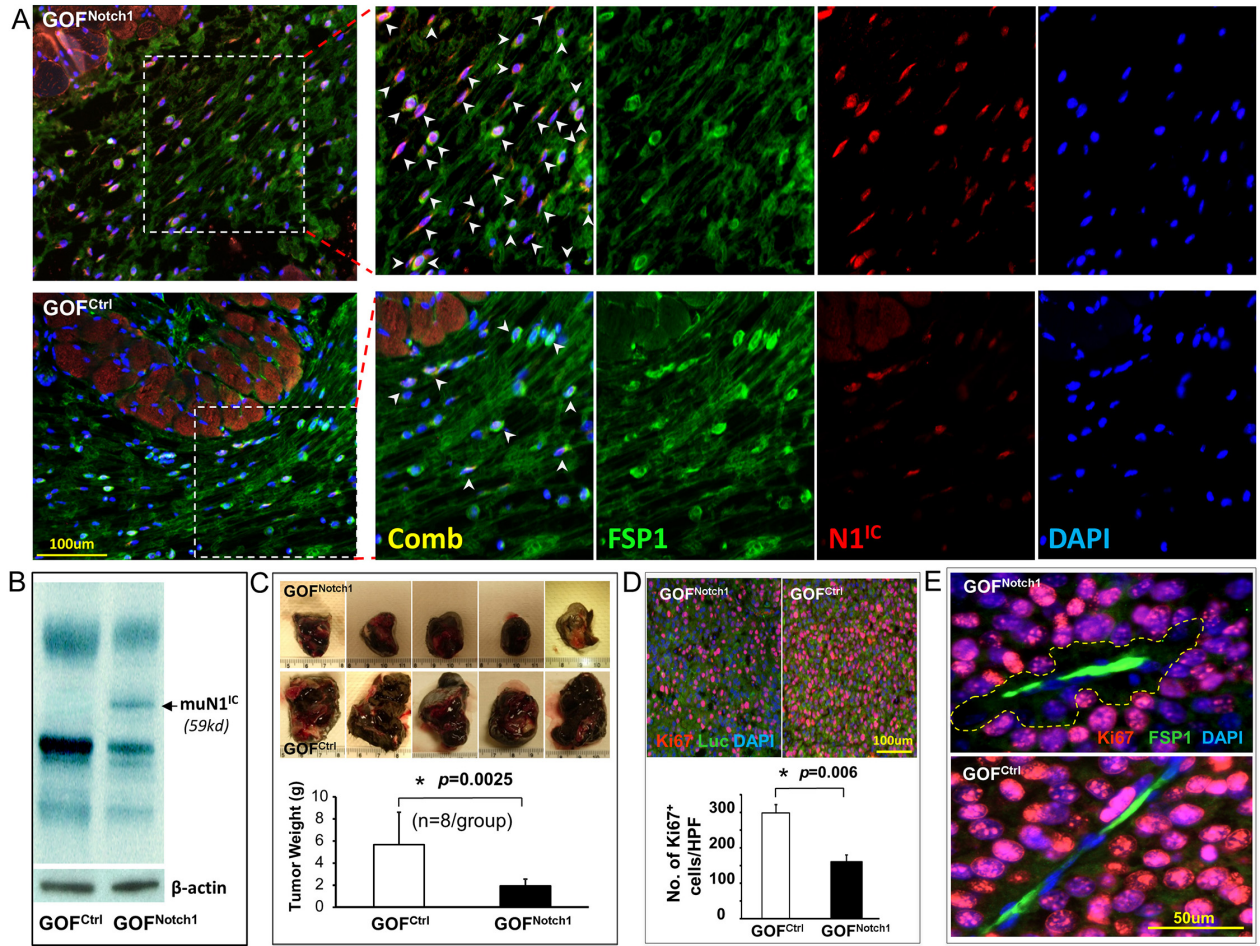
CAF retards melanoma growth in GOF^Notch1^ mice. **A**. Representative images show expression of N1^IC^ in nuclei (arrowheads pointed) of FSP1^+^ skin fibroblasts of GOF^Notch1^ vs. GOF^ctrl^ mice. **B**. Expression of transgene-encoded muN1^IC^ (59 Kd) in skin of GOF^Notch1^ mice was detected by Western blotting. β–actin was used as loading control. **C**. Melanoma growth is retarded in GOF^Notch1^ mice (n = 8/group). Five representative images of resected tumors/group are displayed. **D**. Substantially less Ki67^+^ tumor cells (Luc2^+^) per HPF in melanoma from GOF^Notch1^ than GOF^ctrl^ mice. **E**. Proliferative activity of melanoma cells (Ki67^+^) is particularly low in the area (marked) adjacent to CAF (FSP1^+^) in GOF^Notch1^ mice. Quantifications are counts from 5 HPF per section and 5 section/tumor. The data were statistically analyzed using two-tailed Student’s *t*-test and expressed as the mean ± SD.

IF staining of skin sections portrayed confined and elevated expression of N1^IC^ in nuclei of FSP1^+^ fibroblasts of GOF^Notch1^ mice compared with GOF^ctrl^ mice (Fig 1A), but not in adjacent skin muscle cells (notably muscle cells presented autofluorescence yet no nuclear signals were detectable). Similarly, Hey1 expression was increased in nuclei of FSP1^+^ skin fibroblasts of GOF^Notch1^ mice relative to GOF^ctrl^ mice (S1B Fig). Moreover, the expression of transgene-encoded mutant N1^IC^ protein (muN1^IC^: PEST domain-truncated N1^IC^, 59 Kd, (*ROSA* ^*LSL-N1IC+/+*^, The Jackson Lab: #008159)) in skin of GOF^Notch1^ mice was confirmed by Western blotting (Fig 1B). These results demonstrated efficient Cre-mediated specific expression of N1^IC^ and Notch pathway activation in skin fibroblasts.

Melanoma growth in GOF^Notch1^ mice was significantly retarded in comparison to that in GOF^ctrl^ mice (Fig 1C). Consistently, there were substantially less Ki67^+^ tumor cells (Luc2^+^) in melanoma tissues from GOF^Notch1^ than from GOF^ctrl^ mice (Fig 1D). Proliferative activity (Ki67 positivity) of melanoma cells was particularly weaker when adjacent to CAF (Fig 1E), suggesting that CAF carrying high Notch1 activity in GOF^Notch1^ mice might release tumor suppressive factor(s). These results demonstrated that Notch1 activation confers a tumor-suppressive phenotype on CAF.

### Notch1 activation in CAF suppresses melanoma invasion

**Fig 2:**
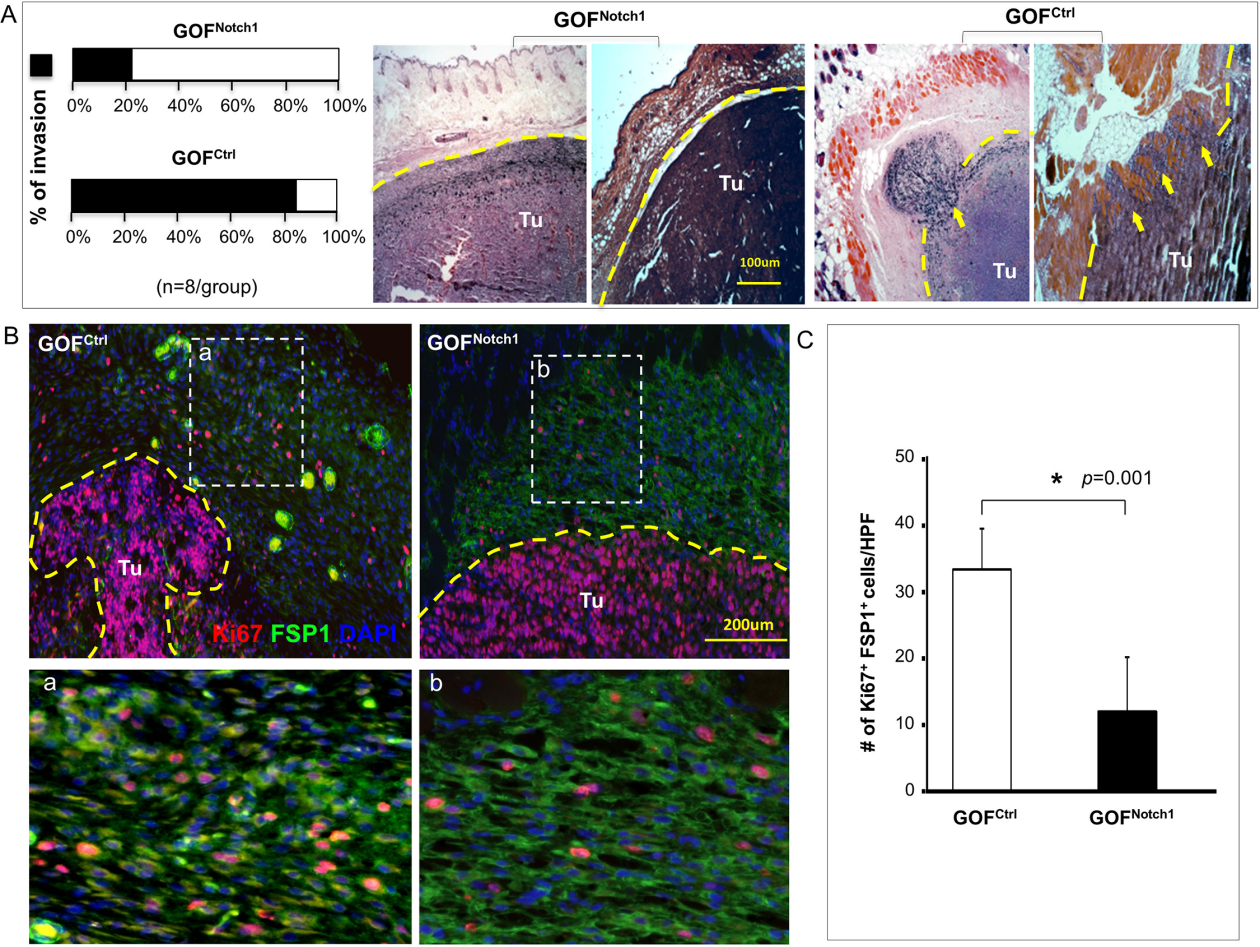
CAF inhibit melanoma invasion in GOF^Notch1^ mice. **A**. *Left*: decreased tumor invasion in GOF^Notch1^ compared with GOF^ctrl^ mice. *Right*: two representative H&E images of tumor sections (n = 8/group). Dash lines indicate tumor boundaries. Arrows point to invading tumor cells. Percentage of invasion is based on results of H&E staining in low power fields (LPF) of each tumor section. **B**. Fibroblasts in the melanoma capsule have lower proliferative activity (less Ki67^+^/FSP1^+^ cells) in GOF^Notch1^ than in GOF^ctrl^ mice. **C**. Quantification of Ki67^+^ fibroblasts/HPF in the melanoma capsule of GOF^Notch1^ (black bar) vs. GOF^ctrl^ (white bar) mice. Results are counts from 5 HPF per section and 5 section/tumor. The data were analyzed using two-tailed Student’s *t*-test and expressed as the mean ± SD.

Since spreading melanoma cells must interact with fibroblasts located in the skin dermis (and in the vicinity of engrafted melanoma), fibroblasts may have a significant impact on melanoma dissemination. We further studied the effect of CAF on melanoma invasion and metastasis in GOF^Notch1^ mice. Melanoma local invasion was evaluated by histological assessment of tissue sections of resected melanoma. H&E staining of resected melanoma tissues illustrated that 83.3% of melanoma had local invasion into adjacent skin tissues in GOF^ctrl^ mice compared with 22.2% in GOF^Notch1^ mice (Fig 2A). However, IVIS scan of whole-body and harvested organs, including lung, liver, spleen, kidney and femur, did not yield measurable luminescent signals (data not shown), which suggest that no distant metastasis occurred at the time of our assay. IF revealed that CAF in tumor capsule displayed lower proliferative activity (less Ki67^+^/FSP1^+^ cells) in GOF^Notch1^ than in GOF^ctrl^ mice (Fig 2B & 2C). These data indicate that CAF in GOF^Notch1^ mice are less supportive to melanoma invasion.

### Null *Notch1* in CAF does not affect melanoma growth

To study the role of CAF with null Notch1 in regulating melanoma growth and invasion, we created the 2^nd^ pair of LOF^Notch1^ vs. LOF^ctrl^ mice. LOF^Notch1^ mice were also viable throughout the melanoma skin graft experiments and their body appearance and skin tissue histology appeared normal (S2A Fig).

**Fig 3:**
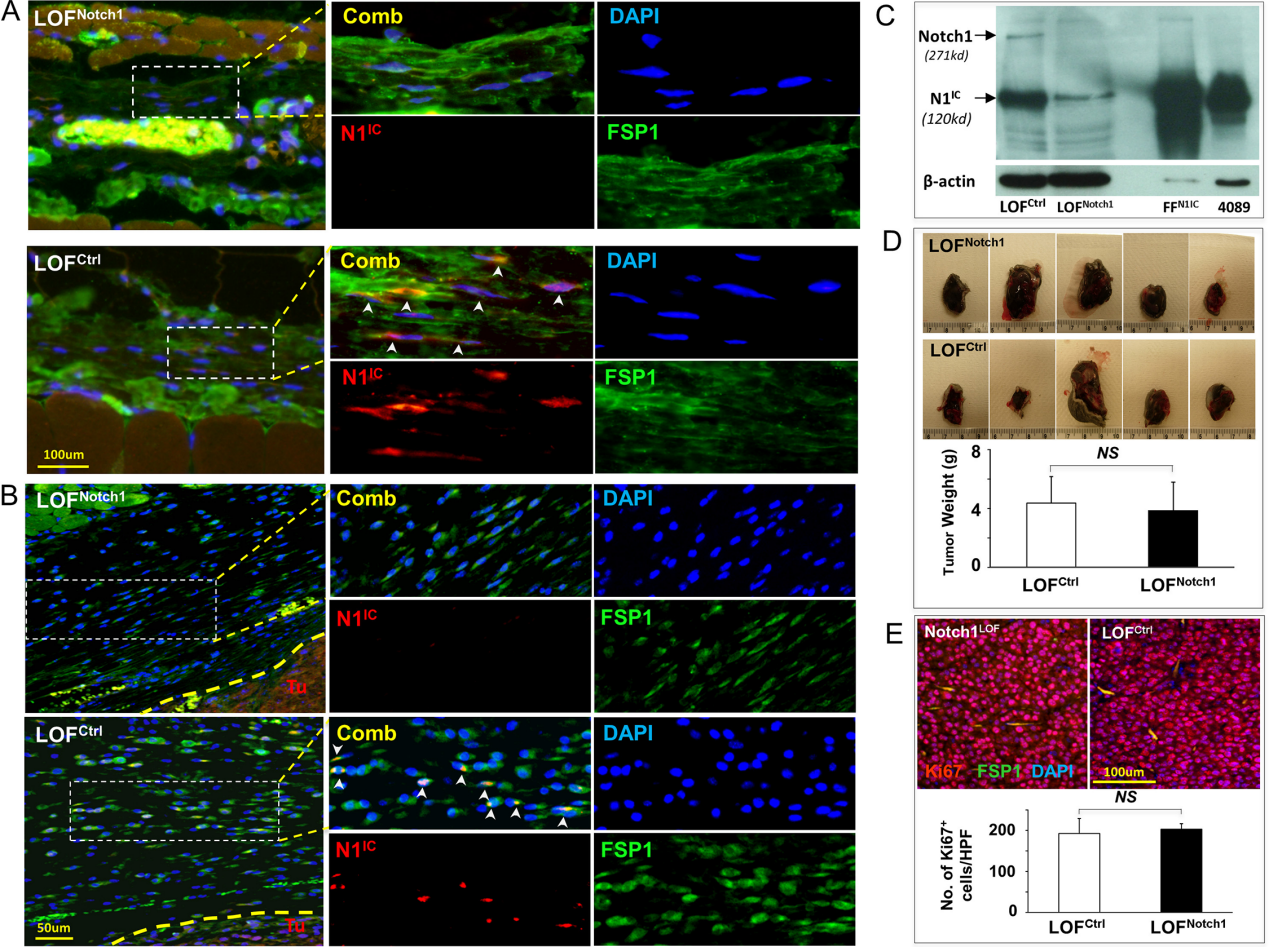
CAF do not affect melanoma growth in LOF^Notch1^ mice. **A**. Undetectable vs. marginally detectable N1^IC^ expression in skin fibroblasts of LOF^Notch1^ vs. LOF^ctrl^ mice. Arrowheads point to nuclear staining of N1^IC^ in fibroblasts. **B**. Undetectable vs. detectable N1^IC^ as pointed by arrowheads in fibroblasts at melanoma capsule in LOF^Notch1^ vs. LOF^ctrl^ mice. **C**. Undetectable full-length of Notch1 protein (271Kd) and slight amount of N1^IC^ (120 Kd, which was likely presented in non-fibroblasts) in skin tissue of LOF^Notch1^ mice was exhibited by Western blotting analysis. β–actin was used as loading control. **D**. Melanoma growth is comparable in LOF^Notch1^ and LOF^ctrl^ mice (n = 8/group). Five representative images of tumors/group are showed. **E**. Numbers of Ki67^+^ tumor cells are comparable in LOF^Notch1^ (black bar) and LOF^ctrl^ (white bar) mice. Quantifications are counts from 5 HPF per section and 5 section/tumor. The data were statistically analyzed using two-tailed Student’s *t*-test and expressed as the mean ± SD.

N1^IC^ and Hes1 were undetectable in FSP1^+^ skin fibroblasts of LOF^Notch1^ mice while slightly detectable in skin of LOF^ctrl^ mice (Fig 3A, S2B Fig). N1^IC^ was also undetectable in fibroblasts at melanoma capsule in LOF^Notch1^ mice (melanoma cells express high levels of N1^IC^ as previously reported [13], serving as an internal control for N1^IC^ expression), but marginally detectable in LOF^ctrl^ mice (Fig 3B). These data indicate the inactivated status of the Notch1 signaling caused by *Notch1* deletion in fibroblasts of LOF^Notch1^ mice vs. basal level status of Notch1 signaling in fibroblasts of LOF^ctrl^ mice. In addition, Western blotting analysis validated successful deletion of Notch1 in skin fibroblasts of LOF^Notch1^ mice. Expression of full-length of Notch1 (271Kd) was undetectable in LOF^Notch1^ mice, although there was slight presence of N1^IC^ (120Kd) (Fig 3C). The marginal levels of N1^IC^ in skin of LOF^ctrl^ mice were ascribed to the presence of N1^IC^ in non-fibroblasts in skin tissue.

To investigate the role of CAF with low or none Notch1 activity in regulating melanoma progression, 5 x 10^5^ Luc2^+^/B16-F10 cells were inoculated (*s*.*c*.) on dorsal skin of 8-week old LOF^Notch1^ vs. LOF^ctrl^ mice. Melanoma skin graft and measurement of tumor growth, invasion and metastasis were conducted identically as described above. The sizes of tumor grafts resected from LOF^Notch1^ are comparable to that from LOF^ctrl^ mice (Fig 3D). Consistently, there was no significant difference in numbers of Ki67^+^ tumor cells (Luc2^+^) within melanoma tissues (Fig 3E) in LOF^Notch1^ vs. LOF^ctrl^ mice. These results showed that CAF in LOF^Notch1^ mice has little effect on melanoma skin growth.

### CAF promotes melanoma invasion in LOF^Notch1^ mice

**Fig 4:**
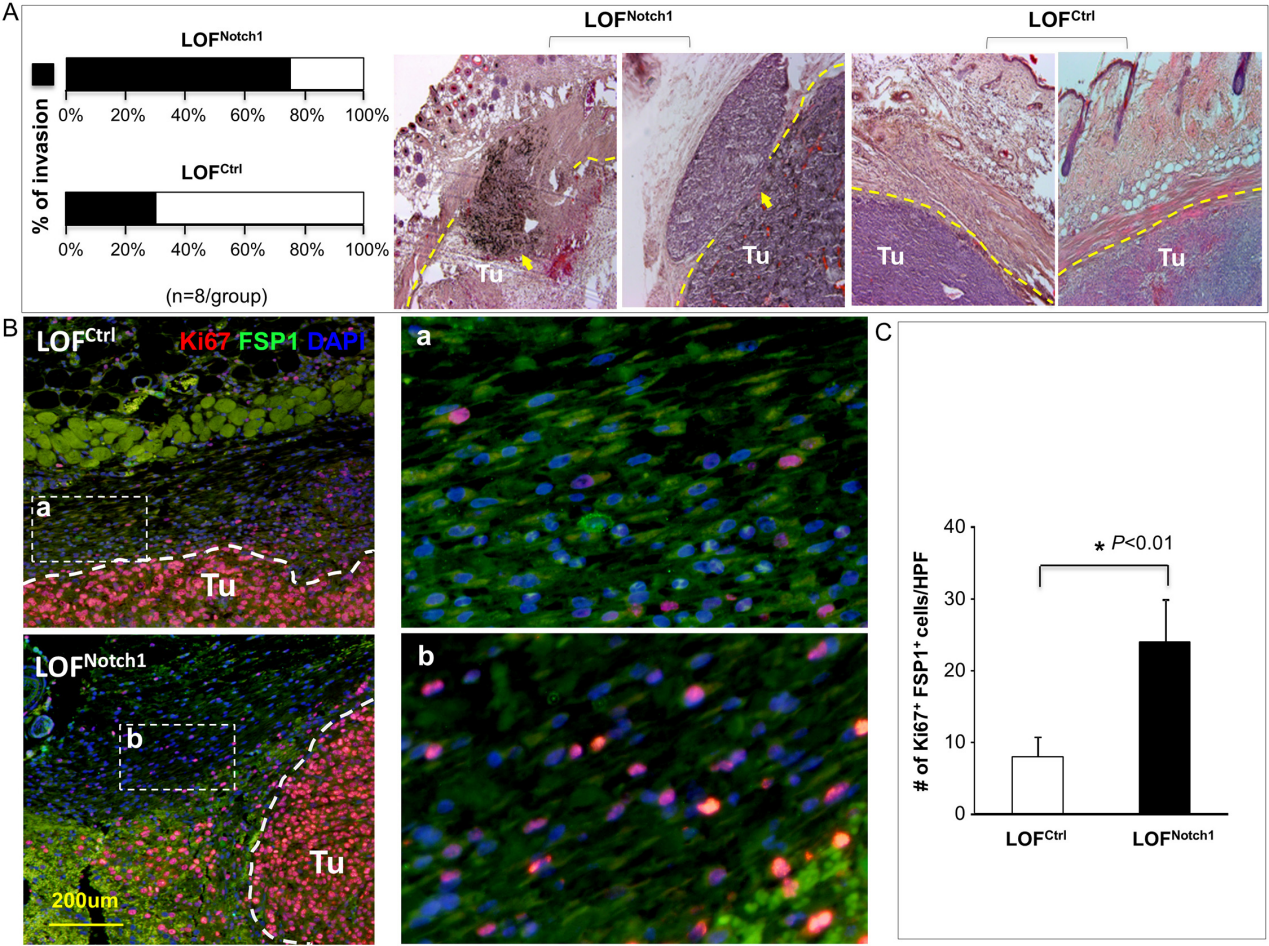
CAF promote melanoma invasion in LOF^Notch1^ mice. **A**. *Left*: Increased melanoma invasion in skin of LOF^Notch1^ vs. LOF^ctrl^ mice (n = 8/group). *Right*: two representative H&E images of tumor sections per group are displayed. Dash lines indicate tumor boundaries. Arrows point to invading tumor cells. Percentage of invasion is based on results of H&E staining in LPF of each tumor section. **B**. Null Notch1 CAF which surround tumors exhibit higher proliferative activity as more Ki67^+^/FSP1^+^ cells are detectable in LOF^Notch1^ than in LOF^ctrl^ mice. **C**. Quantification of Ki67^+^ fibroblasts/HPF in melanoma capsule of LOF^Notch1^ (black bar) vs. LOF^ctrl^ (white bar) mice. Quantifications are counts from 5 HPF per section and 5 section/tumor. The data were statistically analyzed using two-tailed Student’s *t*-test and expressed as the mean ± SD.

In contrast, engrafted melanoma had increased local invasion rates in LOF^Notch1^ (75%) mice than in LOF^ctrl^ (30%) mice (Fig 4A) as evaluated by histological assessment of tissue sections of resected tumor, indicating that CAF promote melanoma invasion in LOF^Notch1^ mice. Consistently, fibroblasts surrounding the tumor in LOF^Notch1^ mice exhibited stronger activity (more Ki67^+^/FSP1^+^ cells) than that in LOF^ctrl^ mice (Fig 4B), suggesting that CAF are more active and may favor melanoma invasion in LOF^Notch1^ mice. Not surprisingly, no distant metastasis was detectable in both sets of mice (data not shown), since the incidence of spontaneous metastasis of grafted B16 cells is very low in the syngeneic murine melanoma model. It typically needs resection of the primary tumor in order for formation of distant metastases to occur [19]. Alternatively, it may be insufficient for a full course of metastasis to be completed within the time frame (3-weeks) of our experiments. Overall, our results demonstrate that deletion of *Notch1* in CAF enhances their regulatory effect on melanoma invasion.

## Discussion

Grafting B16-F10 cells onto skin of GOF^Notch1^ and LOF^Notch1^ mice offer unique syngeneic murine melanoma models for deciphering role of Notch1 signaling in governing tumor-regulating function of CAF in TME in which the entire cellular components of tumor stroma are composed of natural host cells. Our results defined Notch1 signaling as a molecular switch controlling tumor-regulating function of CAF. Turning this molecular switch ON and OFF can inversely confer tumor-suppressive and tumor-promoting properties on CAF. Hence, Notch signaling may be manipulated to implement Notch signaling-directed therapy for melanoma, and potentially other solid tumors. It thus opens a new avenue to target TME by either reprograming and converting CAF from ‘tumor promoters’ to ‘tumor suppressors’ through therapeutic activation of Notch1 pathway or directly exploiting Notch downstream mediator(s), i.e. WISP1 [11]. Although there are numerous options for activation of Notch1 pathway in CAF, such as using gene therapy approach or novel genome editing method, CRISPR/Cas9 or CRISPR/Cpf1, to introduce *N1* ^*IC*^, or applying Notch pathway activating compound, which can be identified through a similar high-throughput screening method [20], activating Notch1 signaling specifically in CAF, while not simultaneously increasing the Notch activity in melanoma cells, pose a therapeutic challenge, as the biological function of Notch signaling is cell context-dependent [21], and high Notch activity is oncogenic to melanoma [13]. It is unclear how Notch signaling is differentially regulated in CAF and melanoma cells in a single microenvironment. It also remains unclear whether Notch/ligands participate in cell-cell communication between fibroblasts-melanoma cells. Since a significant fraction of CAF in tumor tissue are derived from mesenchymal stem cells (MSC), an alternative strategy is to develop cell-based therapies by targeted delivery of therapeutic cells, i.e. autologous MSC-derived fibroblasts pre-engineered ‘*ex vivo*’ to either carry high Notch1 activity using methods described above or overexpress WISP1, into tumor tissue. Fibroblasts expressing high Notch activity tend to undergo cell cycle arrest [9,10]. This character makes MSCD-SF carrying high Notch activity more appealing as therapeutic cells, because they will not expand irresistibly after their homing to tumor tissue and are eventually cleared by immune cells. Therefore, they can be repeatedly applied to patients to enhance therapeutic efficacy.

Unlike the inhibitory effects of CAF in GOF^Notch1^ mice on both melanoma growth and invasion, CAF in LOF^Notch1^ mice promote local invasion, but not skin growth of melanoma. The mechanism for such distinct effects of CAF in GOF^Notch1^ mice vs. LOF^Notch1^ mice remains unclear. Possibly, the growth and invasion properties of melanoma are regulated by different soluble factors and microenvironmental cues created by CAF. Deletion of *Notch1* in CAF may result in change of a set of soluble factors and microenvironmental cues, which preferentially or sufficiently affects melanoma invasion, but not growth property. On the other hand, soluble factors and microenvironmental cues created by CAF, which determine melanoma growth property, may not be exactly inverted between GOF^Notch1^ and LOF^Notch1^ mice. The Notch1 pathway is hyper-activated through expression of N1^IC^ mutant in CAF of GOF^Notch1^ mice, which is different from canonical, ligand-induced Notch1 activation where deletion of Notch1 may inversely mirror. This could explain why CAF in GOF^Notch1^ mice retarded melanoma growth, while CAF in LOF^Notch1^ mice do not promote melanoma growth. Alternatively, other Notch isoforms may compensate for Notch1 loss in CAF of LOF^Notch1^ mice. Future studies are warranted to examine complete profiles of soluble factors and microenvironmental cues determined by different CAF (CAF from GOF^Notch1^ mice vs. CAF from GOF^Ctrl^ mice and CAF from LOF^Notch1^ mice vs. CAF from LOF^Ctrl^ mice).

Another interesting, yet unexplained finding is the distinct spontaneous invasion rates of B16-F10 cells grafted on GOF^Ctrl^ (83.3%) vs. LOF^Ctrl^ (30%) mice, possibly due to the different genetic backgrounds of two lines of mice. MSC-derived fibroblasts from these two lines of control mice also exhibit different tumor-regulating phenotypes *in vitro*. Fibroblasts from GOF^Ctrl^ mice induced melanoma cells to form spheroids while those from LOF^Ctrl^ mice did not (unpublished observation).

In summary, we uncover the Notch1 pathway as a molecular determinant that controls the regulatory role of CAF in melanoma growth and invasion. Our study highlights the Notch1 pathway as a potential therapeutic target to be manipulated to reprogram and convert CAF to act as tumor suppressors. These findings warrant future study to elucidate molecular mechanisms for the Notch1-determined tumor-regulating role in CAF.

## Supporting Information

S1 Fig **A,** Representative appearance pictures of GOFNotch1 and GOFCtrl. Skin tissue histology appears normal as examined by H&E at week 6. **B,** Elevated expression of Hey1 in skin fibroblasts of GOFNotch1 mice compared with GOFCtrl mice. Arrowheads point to nuclear-localized Hey1 in fibroblasts. Antibody recognizes Hey-1 was purchased from GeneTex (GTX42614).
(PDF)

S2 Fig **A,** Representative appearance pictures of LOFNotch1 and LOFCtrl. Skin tissue histology appears normal as examined by H&E at week 6. **B,** Hes1 expression is undetectable in fibroblasts located at capsule of melanoma in LOFNotch1 mice but slightly detectable at capsule of melanoma LOFCtrl mice. Arrowheads point to nuclear-localized Hes1 in fibroblasts. Antibody recognizes Hes1 was purchased from Abcam (ab71559).
(PDF)
