# Supplementary material for: Notch1 Pathway Activity Determines the Regulatory Role of Cancer-Associated Fibroblasts in Melanoma Growth and Invasion
Source: PLoS One. 2015 Nov 12;10(11):e0142815. doi: 10.1371/journal.pone.0142815 (PMC4643021; doi:10.1371/journal.pone.0142815)

S1 Fig. A. Representative appearance pictures of GOF^{Notch1} and GOF^{Ctrl}. Skin tissue histology appears normal as examined by H&E at week 6. **B**. Elevated expression of Hey1 in skin fibroblasts of GOF^{Notch1} mice compared with GOF^{Ctrl} mice. Arrowheads point to nuclear-localized Hey1 in fibroblasts. Antibody recognizes Hey-1 was purchased from GeneTex (GTX42614).

Suppl. Figure 1

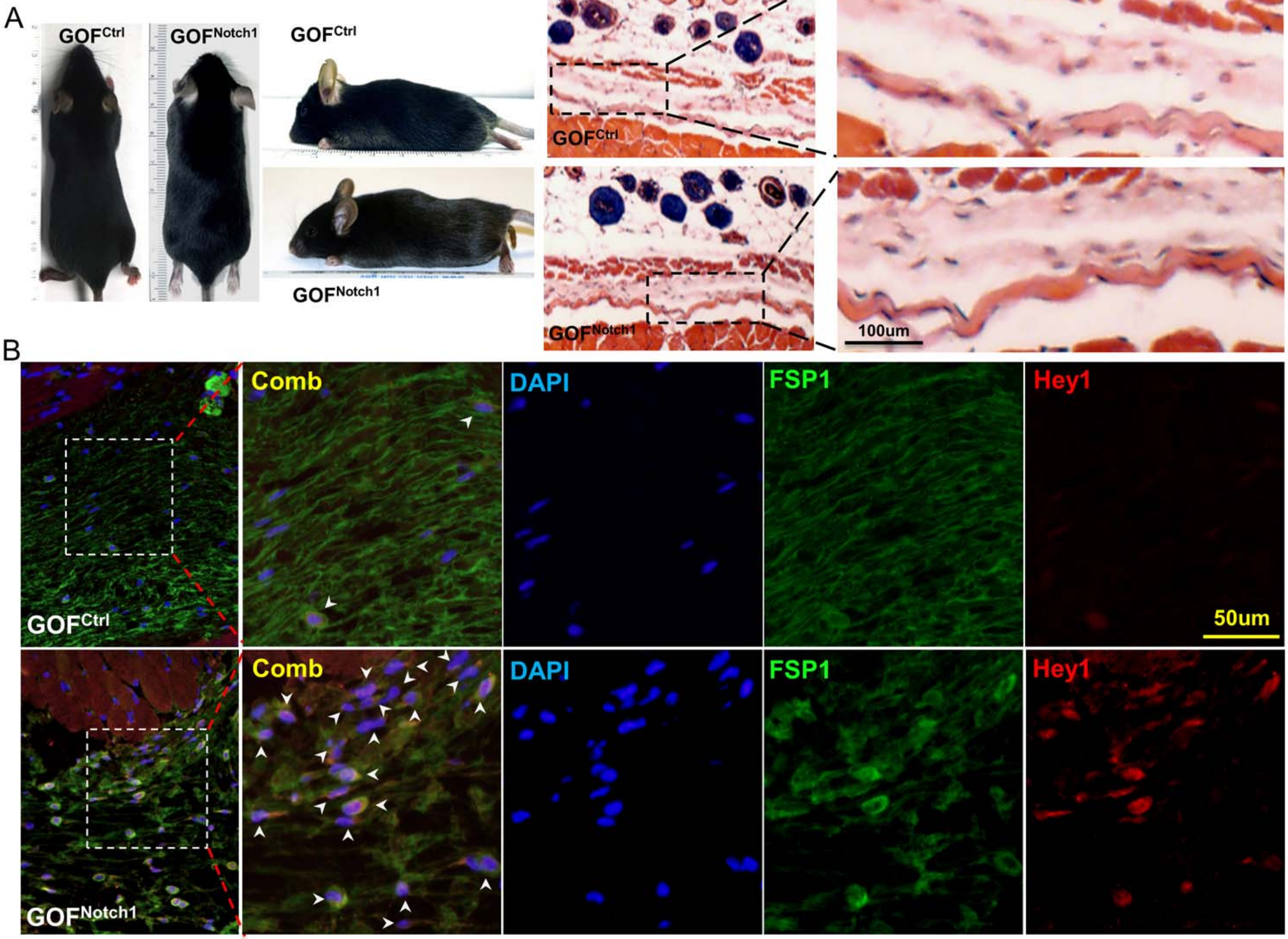

Supplement: S1 Fig — A, Representative appearance pictures of GOFNotch1 and GOFCtrl. Skin tissue histology appears normal as examined by H&E at week 6. B, Elevated expression of Hey1 in skin fibroblasts of GOFNotch1 mice compared with GOFCtrl mice. Arrowheads point to nuclear-localized Hey1 in fibroblasts. Antibody recognizes Hey-1 was purchased from GeneTex (GTX42614). (PDF) [file pone.0142815.s001.pdf]
