# Supplementary material for: Notch1 Pathway Activity Determines the Regulatory Role of Cancer-Associated Fibroblasts in Melanoma Growth and Invasion
Source: PLoS One. 2015 Nov 12;10(11):e0142815. doi: 10.1371/journal.pone.0142815 (PMC4643021; doi:10.1371/journal.pone.0142815)

S2 Fig. A. Representative appearance pictures of LOF^{Notch1} and LOF^{Ctrl}. Skin tissue histology appears normal as examined by H&E at week 6. **B**. Hes1 expression is undetectable in fibroblasts located at capsule of melanoma in LOF^{Notch1} mice but slightly detectable at capsule of melanoma LOF^{Ctrl} mice. Arrowheads point to nuclear-localized Hes1 in fibroblasts. Antibody recognizes Hes1 was purchased from Abcam (ab71559).

Suppl. Figure 2

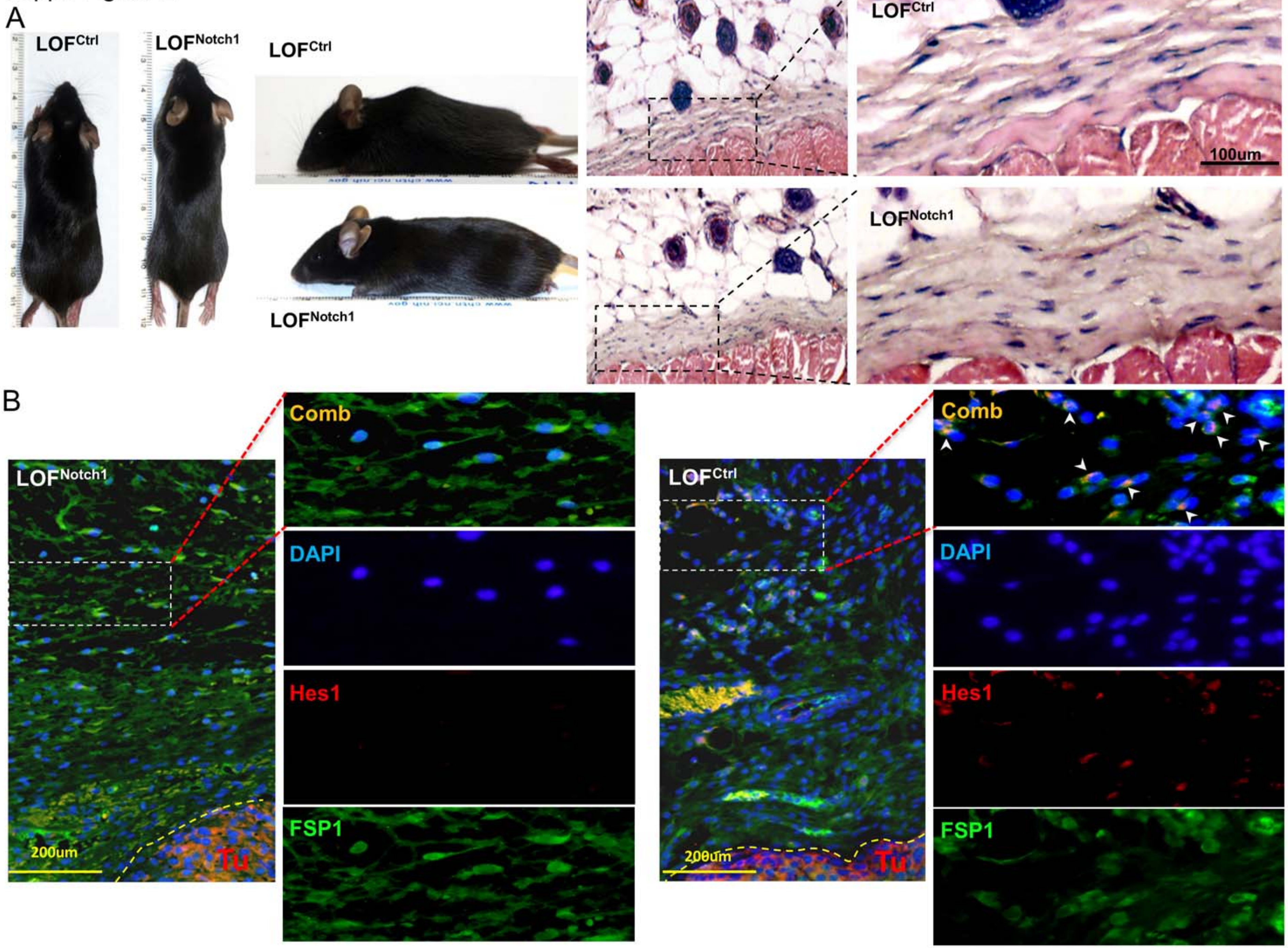

Supplement: S2 Fig — A, Representative appearance pictures of LOFNotch1 and LOFCtrl. Skin tissue histology appears normal as examined by H&E at week 6. B, Hes1 expression is undetectable in fibroblasts located at capsule of melanoma in LOFNotch1 mice but slightly detectable at capsule of melanoma LOFCtrl mice. Arrowheads point to nuclear-localized Hes1 in fibroblasts. Antibody recognizes Hes1 was purchased from Abcam (ab71559). (PDF) [file pone.0142815.s002.pdf]
